# Evaluation of the Emergency Obstetric and Newborn Care training in Gondar, Ethiopia; a mixed methods study

**DOI:** 10.1371/journal.pgph.0000889

**Published:** 2023-09-26

**Authors:** Myrrith Hulsbergen, Birhanu Abera, Mulat Adefris, Dawit Kassahun, Marieke Meulenbeld, Sabine van Nievelt, Charles Ameh, Mimosa Bruinooge, Marcus J. Rijken, Jelle Stekelenburg

**Affiliations:** 1 Working Party on International Safe Motherhood and Reproductive Health, Groningen, Netherlands; 2 Center for Evidence Based Education, Amsterdam University Medical Center, Amsterdam, The Netherlands; 3 Gynaecology Department, Women’s Healthcare Center, Amsterdam, The Netherlands; 4 Department of Obstetrics and Gynecology, University of Global Health Equity (UGHE), Kigali, Rwanda; 5 Department of Obstetrics and Gynaecology, University of Gondar, Gondar, Ethiopia; 6 Department of Obstetrics and Gynaecology, Haaglanden Medical Centre, Hague, The Netherlands; 7 International Public Health Department Liverpool School of Tropical Medicine, Emergency Obstetric Care and Quality of Care Unit, Liverpool, United Kingdom; 8 Department of Obstetrics and Gynaecology, Admiraal de Ruyter Ziekenhuis, Goes, The Netherlands; 9 Julius Global Health, Julius Centre for Health Sciences and Primary Care, University Medical Centre Utrecht, University of Utrecht, Utrecht, The Netherlands; 10 Department of Obstetrics and Gynaecology, Leeuwarden Medical Centre, Leeuwarden, The Netherlands; 11 Department of Health Sciences, Global Health, University Medical Centre Groningen/University of Groningen, Groningen, The Netherlands; PLOS: Public Library of Science, UNITED STATES

## Abstract

In Ethiopia maternal and perinatal morbidity and mortality remains high. Timely access to quality emergency obstetric and neonatal care is essential for the prevention of adverse outcomes. Training healthcare providers can play an important role in improving quality of care, thereby reducing maternal and perinatal mortality and morbidity. The aim of this study was to evaluate change of knowledge, skills and behaviour in health workers who attended a postgraduate Emergency Obstetric and Newborn Care training in Gondar, Ethiopia. A descriptive study with before-after approach, using a mix of quantitative and qualitative data, based on Kirkpatrick’s model for training evaluation was conducted. The evaluation focussed on reaction, knowledge, skills, and change in behaviour in clinical practice of health care providers and facilitator’s perspectives on performance. A ‘lessons learned approach’ was included to summarize facilitators’ perspectives. Health care providers reacted positively to the Emergency Obstetric and Newborn Care training with significant improvement in knowledge and skills. Of the 56 participants who attended the training, 44 (79%) were midwives. The main evaluation score for lectures was 4,51 (SD 0,19) and for breakout sessions was 4,52 (SD 0.18) on scale of 1–5. There was a statistically significant difference in the pre and post knowledge (n = 28, mean difference 13.8%, SD 13.5, t = 6.216, p<0.001) and skills assessments (n = 23, mean difference 27.4%, SD 22.1%, t = 5.941, p<0.001). The results were the same for every component of the skills and knowledge assessment. Overall, they felt more confident in performing skills after being trained. Local sustainability, participant commitment and local context were identified as challenging factors after introducing a new training program. In Gondar Ethiopia, the Emergency Obstetric and Newborn Care training has the potential to increase skilled attendance at birth and improve quality of care, both vital to the reduction of maternal and perinatal mortality and morbidity.

## Introduction

Every day, approximately 810 women die from preventable causes related to pregnancy and childbirth [1]. Sixty-six per cent of the world’s maternal deaths occurs in sub-Saharan Africa, which hosts 11% of the global population [2–4]. With a Maternal Mortality Ratio (MMR) of 401 per 100.000 live births, Ethiopia substantially contributes to this number [5]. As most maternal deaths occur during labour, delivery and the first day postpartum, it is particularly important that all births are attended by skilled health professionals [6,7]. In 2016, only 28% of pregnant women delivered with a skilled birth attendant (SBA) in Ethiopia [2,8]. So far, approaches used to improve coverage of SBA in Ethiopia were aimed to ensure the availability and accessibility of quality Emergency Obstetric and Neonatal Care (EmONC) services. In response to a national EmONC assessment, in 2010 the Ethiopian government developed a national Basic Emergency Obstetric and Neonatal Care (BEmONC) 21-day in-service training for midwives and nurses [9].

The Liverpool School of Tropical Medicine (LSTM) EmONC training is a short multidisciplinary, comprehensive, competency based, in-service training which had a significant positive impact on participant knowledge and skills in several African and Asian countries [10–13]. In 2015, the Working Party International Safe Motherhood and Reproductive Health (WP ISM & RH) introduced this training in Gondar Ethiopia in response to a request from one of our collaborating partners, World Vision Ethiopia. Despite the earlier introduction of the 21-day in-service BEmONC training in Gondar, there was a perceived insufficient quality of care in the maternity ward of Gondar University Hospital and in the health centers in and around Gondar. This resulted in the request for a short course training as given by the LSTM. The newly developed blended 12-day BEmONC course was only introduced in 2017–2018 [14].

This study aimed to evaluate change of knowledge, skills and behaviour in health workers who attended the LSTM-EmONC training and to attain lessons learned from introducing the training in the setting of Gondar, Ethiopia. With the lessons learned approach, this study included a new approach to training evaluation, which in general might be useful for a wider context of introducing training programs in low-income settings.

## Methods

In June 2015 and February 2016 skilled health care providers from health centres in Gondar with a referral linkage to the teaching hospital participated in the LSTM-EmONC training [10–13,15]. This was part of a collaborative project between the Dutch Working Party on ‘International Safe Motherhood and Reproductive Health’, World Vision Ethiopia, LSTM and the Department of Obstetrics and Gynaecology, University of Gondar. This training was developed in 2006 to increase the quality of skilled birth attendants in the recognition and treatment of maternal complications, in order to reduce maternal mortality. Extensive description of the rational for developing and adapting the LSTM EmOC training for various settings is extensively presented elsewhere [13]. The LSTM- EmONC training is a standardized 3.5-day training using a mixture of learning methods including lectures, skills training, scenario teaching, workshops, demonstrations and discussions [11]. The course is designed to cover the five major causes of maternal death (haemorrhage, sepsis, eclampsia, obstructed labour and unsafe abortion), newborn resuscitation and early newborn care. Led by a course director and a group of facilitators, all participants practice skills in an interactive manner in small groups (breakout sessions). During the first training these facilitators were gynaecologists from the Netherlands who completed the LSTM Training of Trainers training provided by the LSTM [11]. In a joined effort, the LSTM-EmONC training was aligned as much as possible with the local Ethiopian obstetric guidelines. Eight Ethiopian gynaecologists and residents were trained, identified and certified as facilitators for the following trainings. All trainers had participated in the training previously; they received a special training for trainers of the course and passed an assessment to qualify as trainers. All scoring systems were standardized to minimize the risk of interobserver bias. Gondar University Hospital has a training setting spacious enough with one lecture hall and several breakout rooms to organize the LSTM -EmONC training. Equipment was taken from Liverpool during the 1^st^ training; in the following trainings equipment from GUH was used, partly supplemented with materials from the Netherlands.

### Study design

This was a descriptive study in which pre and post quantitative (assessments) and qualitative (semi-structured interviews) data from the training were collected (Table 1). An adapted framework of Kirkpatrick’s (KP) model, an internationally recognized tool for evaluating training programs was used [13,16]. The assessment included: 1) evaluation of the impact of the training on knowledge and skills (KP 1&2), 2) evaluation of the impact of the training on behaviour in clinical practice (KP 3), and we added 3) a lessons learned approach.

**Table 1:** Summary of methodology for evaluation of LSTM-EmONC training in Gondar, 2015–2016.

	Description	Method	Scale
**Kirkpatrick Level 1:** **Reaction**	Participant reaction to all aspects of training using anonymous self-administered questionnaires.	Evaluation during the training. Participants were regularly reminded during the training to score each training activity based on their satisfaction.	Response scale ranging from not useful/enjoyable (1) till extremely useful/enjoyable (5)
**Kirkpatrick Level 2:** **Knowledge & skills**	Standardized multiple-choice and open questions and skills check lists. Skills assessments were carried out using obstetric, newborn and life-saving skill mannequins.	All participants were involved in a knowledge test and participated in skills assessments before and after the training.	Knowledge test: 26 true or false questions with maximum (failure) score of 26. Skills test: 3 sections (basic life support of newborn; manual vacuum aspiration; breech delivery) of each 20 points.
**Kirkpatrick Level 3:** **Behaviour**	Qualitative assessment focussed on change in behaviour by using interviews with trainees of 7 different health centres 3–6 months after training.	Participants were interviewed during follow-up visit 3 months after training. Facility inventory was done during the health centre visit.	Scale of confidence from no confidence (1) till performing independently with confidence (3).
**Lessons learned**	Qualitative assessment using interviews, inspections and observations.	Facilitators were interviewed.	Facilitators’ experiences during the training and at the health centre visits were summarized and structured into lessons learned themes

Anonymous self-administered questionnaires consisting of three sections (feedback on lectures, feedback on breakout sessions, overall feedback) were used to assess the participant reaction (KP 1) to the training. The participants scored the activities of these sections on a 5-point response scale ranging from not useful/enjoyable (score = 1) to extremely useful/enjoyable (score = 5).

The knowledge of the participants was assessed using 46 multiple choice questions covering post-partum haemorrhage (PPH), pre-eclampsia/eclampsia, assisted vaginal birth and obstructed labour. Each component assessed was scored out of 100 percent, the mean score and standard deviation was also calculated. Each participant was assessed on 2 randomly selected EmOC skill from MVA, newborn resuscitation, and breech vaginal birth. Each skills test was 20 marks and the combined skills score for each participant was calculated out of 100 percent.

All correct responses were scored and calculated to 100% for each participant. We determined the mean percentage score and standard deviation (SD) for each test, combined knowledge test and combined knowledge test. Only complete data sets were included for each participant.

Change in behaviour after training (3 and 6 months) was assessed during supervisory visits at the health centres by collecting data from midwives, delivery registers about change in behaviour and practice, facility preparedness (availability of drugs and supplies), factors enabling or inhibiting the performance of emergency obstetric care in the health centres. A semi-quantitative questionnaire was administered to ten midwives from the surrounding health centres of Gondar University Hospital to assess their level of confidence to perform emergency obstetric and early newborn care on a scale from 1 to 3 (level 1 represented no confidence in performing; level 2 performing under supervision; and level 3 performing independently with confidence). After data collection, the midwives were given additional supervision in form of hands-on skills and simulation-based training on any EmONC topic requested.

Finally, feedback on the training programme was collected to document lessons learned in setting up this training program. Two open questions on 4 pre-defined themes were asked to the ten health center staff during post-training visits and to the key facilitators of the course: “what could have gone better” and “how would you suggest improving this training”. The four themes were: training participation of trainees and facilitators, memorandum of understanding, and follow-up. Answers were manually summarized by the researchers.

### Data processing and statistical analysis

Paired student T test was used to determine the difference in pre- and post-test assessments and a significance P-value was set at <0.05. Statistical Package for Social Sciences (SPSS) version 26 was used to analyse the data.

### Ethics statement

Ethical clearance was obtained from the Institutional Review Board of the University of Gondar, Ethiopia (ref no. 356/2015). Oral consent was taken since this research is about the evaluation of the training itself, and not an evaluation of the trainees. This consent was plenary taken at the beginning of the training and during the health center visits. All training participants (trainees and facilitators) agreed that the results of their test and their evaluation of the training would be summarized and used for analysis. The consent was not documented.

All data were extracted from the questionnaires used during the training and during the health center visits. All information was fully anonymized.

### Inclusivity in global research

Additional information regarding the ethical, cultural, and scientific considerations specific to inclusivity in global research is included in the Supporting Information (SX Checklist).

## Results

### Participants

A total of 56 participants attended the LSTM-EmONC training in June 2015 and February 2016 (Table 2). Six of the participants from GUH had joined the 21-day BEmONC or the CEmONC training in the past. Most of the participants were midwives (79%).

**Table 2:** Cadres of medical staff trained (n = 56), LSTM-EmONC training in Gondar, 2015–2016.

Cadres of medical staff trained	No. (%)
**Health Centers:**	
Midwife	30 (55,6%)
Health officer	1 (1,9%)
**Gondar University Hospital:**	
Midwife	14 (25,9%)
Medical doctor, resident	4 (7,4%)
Medical doctor, gynaecologist	4 (7,4%)
Medical student	1 (1,9%)
Unknown function	2 (3.6%)
**Total:**	56 (100%)

### Feedback on the training

The overall response rate for the participant feedback form was 89% (n = 50). The scale ranging from not useful/enjoyable (1) till extremely useful/enjoyable (5), showed that the mean score for all lectures was 4.51 (SD 0,19), with the highest score (4,79) on lecture ‘perineal repair and manual vacuum aspiration’ and the lowest score (4,38) on lecture ‘shock’. The mean score for the breakout sessions was 4,52 (SD 0,18)with the highest score on the breakout sessions of day 3 of the training (breech delivery, shoulder dystocia, cord prolapse, twin delivery). The breakout sessions on day 1 (airway and resuscitation, newborn care, venous cut down) had a slightly lower score 4,37. The overall feedback showed that the participants scored the training as being enjoyable 4,5 (SD 0,41) and useful for their job 4,7 (SD 0,53).

Eleven participants provided written feedback, these included suggestions to extend the training, appreciation of the training and suggestions for improvement (S1 Text).

### Gondar training evaluation

There was a statistically significant difference in the pre and post knowledge (n = 28, mean difference 13.8%, SD 13.5, t = 6.216, p<0.001) and skills assessments (n = 23, mean difference 27.4%, SD 22.1%, t = 5.941, p<0.001). The results were the same for every component of the skills and knowledge assessment (Table 3).

**Table 3:** Results of the knowledge and skill stests, LSTM-EmONC training in Gondar, 2015–2016.

	N	Pretest mean (%)	SD (%)	Posttest mean (%)		SD (%)	Mean Difference (%)	SD (%)	t	P-value
**Knowledge tests**										
**Post-partum haemorrhage (PPH)**	27	74.1	15.5	82.9	14.8		-8.8	14.6	-3.124	0.004
**Pre-eclampsia/eclampsia**	26	53.8	28.2	81.3	17.0		-27.4	24.0	-5.826	<0.001
**Assisted vaginal birth**	28	47.0	33.3	64.9	25.0		-17.9	27.9	-3.382	0.002
**Obstructed labour**	28	90.0	10.5	93.1	12.4		-3.1	15.2	-1.075	0.292
**Combined Knowledge assessments**	28	64.6	16.8	80.5	13.6		-15.8	13.5	-6.216	<0.001
**Skills tests**										
**Manual vacuum aspiration (MVA)**	26	65.8	22.1	88.7	14.0		-22.9	20.7	-5.638	<0.001
**Newborn resuscitation**	9	42.2	34.2	81.1	22.5		-38.9	35.6	-3.277	0.011
**Breech**	11	39.1	35.0	75.5	19.7		-36.4	28.6	-4.211	0.002
**Combined Skill assessments**	23	54,9	27.1	82.3	14.1		-27.4	22.1	-5.941	<0.001

### Follow-up after the training

Ten midwives completed the follow-up questionnaire about the training. Most of them (n = 8) felt that they had enough exposure to emergency obstetric cases in the period after the training to remain proficient in the skills, according to their reported self-confidence. However, they requested additional training in neonatal resuscitation, obstetric emergencies, venous cut down, partograph use and shock, perineal tear repair, cord prolapse and manual vacuum aspiration. Overall, after being trained the midwives felt confident to independently perform most skills. The skills they still preferred to perform under supervision were venous cut down; managing obstetric emergencies (breech delivery, twin delivery, shoulder dystocia); cord prolapse, and suturing perineal and cervical tears (S1 Table). Lack of drugs and equipment were the main barriers to performing EmONC (S2 Text). Lack of a reliable referral system was identified as threat to the availability of EmONC: the midwives from all health centres missed but wished feedback from the receiving hospital about the women they had referred, in order to improve the overall quality of care and communication with referral centres.

### Facilitator experiences

Facilitators were asked about their training experiences to identify challenges and suggestions for improvement. Due to misunderstanding about the duration of the course and per diem payment, some of the participants refused to join the training in February 2016, delaying the start of the training by one day. Suggestion for improvement was clear communication using personal invitations for all trainees with all details as mentioned above, whereby the difference between this short course and the standard 21-day BEmONC should be explained. The tight schedule of the training proved to be problematic during every training day. Full commitment of local staff to facilitate the training was hampered by other obligations, such as clinical night shifts, or giving lectures to other students during the training. It was suggested that the facilitators should have no other obligations during the training, and that only residents and midwifes should be facilitating the course. It was advised to develop a Memorandum of Understanding to clearly set the goals and responsibilities of all collaborating parties. Also, suggestions for the content of the training were made: one local facilitator stated: “*topics that could really make a difference could have had more attention during the training*, *for example vacuum extraction compared to cardiopulmonary resuscitation of the adult*”. We found that some trainees participated with the aim to learn advanced obstetric skills but were less interested in the overall content and goal of the training. Lastly, an evaluation plan was considered to ensure that what was being taught was put into practice.

## Discussion

We report the first evaluation of the LSTM-EmONC training in Gondar, Ethiopia based on Kirkpatrick’s model for training evaluation. A significant improvement on participant knowledge and skills (KP 2) was found, which is in line with previous reports from the training evaluation in other low- and middle-income countries [10–13,17,18]. In many settings the LSTM-EmONC training was adapted to suit the specific settings in which it was delivered [13]. Adaptations included for example using WHO modified partograph instead of WHO composite partograph and using additional content such as malaria in pregnancy, HIV in pregnancy and medical ethics.

The LSTM-EmONC training is based on the common direct causes of maternal deaths and consists of mixed interactive and didactic education techniques [11]. This mixture of training techniques has been shown to be effective [19]. Indeed, the midwives and doctors from Gondar University Hospital and surrounding health centres reported a high level of satisfaction with the training (KP 1).

In 2010, the Ethiopian government scaled up the EmONC services by standardizing the BEmONC in-service training of 21 days. However, many of these trainees (40%) did not achieve knowledge-based mastery after the BEmONC training, defined as scores ≥85% [20,21]. In contrary, this study showed that introducing the much shorter LSTM-EmONC training in Gondar did result in improved knowledge and skills. In other studies short-term structured skills training also resulted in significantly improved scores for knowledge and scenario-based practice among participants [22,23]. Moreover, there is evidence that short competency-based EmONC training programs are more effective than longer didactic-based training programs [13]. Furthermore, such short trainings have less compromising impact on service delivery at the health centres because there is no long detachment from work.

In the LSTM-EmONC training reported here, different cadres of trainees were included: gynaecologists, residents and midwives both from Gondar University Hospital and the surrounding health centres. It is evident that among and within these cadres’ heterogeneity existed in knowledge, skills and behaviour at the start of the training based on e.g. experience, capacity, earlier courses and education, etc. Having followed the conventional 21-day course also contributed to this heterogeneity. Since this study aimed to evaluate the effect of the training and applied a before-after approach, stratification was not deemed to be necessary.

The combination of diverse cadres of health care workers in the same team enabled skill training such as communication, referral, triage and providing and receiving feedback. Ineffective teamwork and communication have been proven to contribute to maternal and perinatal mortality [24]. Training in a multidisciplinary team provides opportunities to participants to learn from each other. In a study conducted in Guatemala, mutual respect between midwives and traditional birth attendants contributed to an increased number of referrals, which was seen as an improvement in quality of care [24]. Likewise, during the supervisory visits the midwives brought up that poor referral network is an important problem in Ethiopia. Improving the relationship and communication between staff in health centres and in GUH is thus of great importance. Measuring a change in behaviour (KP 3) in clinical practice is challenging because there may be several confounding factors such as the availability of equipment and essential drugs, the availability of other training programs and the level of facility utilization. To avoid bias due to other training programs in the region, especially in Gondar Hospital, only inexperienced midwives (untrained midwives or recently graduated) were invited to participate in the assessment of change in clinical behaviour (KP 3) after the LSTM-EmONC training. The midwives felt a high level of confidence in performing most of the skills, which may suggest a positive behaviour change in clinical practice (KP 3).

On-going education after training could lead to better scores on skill performance. During the follow-up visits after the training, trainees were provided with any hands-on skills training in topics they still felt insecure about. A study conducted in Indonesia showed that participants receiving peer review and continuing education after skill training had better scores on skill performance, compared to participants not receiving any follow-up [18]. This emphasizes the importance of supervisory follow-up visits and regularly mentoring of the trainees after the introduction of an EmONC training.

The reported challenges concerned the availability of supplies, of trainees and trainers. Firstly, the venue was suitable to give the training, but the availability of mannequins and supplies could have been improved. Problems were solved, whereby WorldVision was responsible of the smaller supplies and the WP ISM & RH brought some materials as well. Secondly, trainees were not used to the strict timing of start and continuation of the course and local trainers had difficulty in keeping time due to other activities in the hospital or due to private issues. Thirdly, financial compensation for the local trainers and incentives for the trainees were a challenge as well, which were covered by both the WP ISM & RH and WorldVision. These challenges on attendance and payments will possibly be limited by ensuring local ownership of the training. Also, the use of a Memorandum of Understanding with clear agreements on payment and presence during the course will help to improve the availability of Ethiopian trainers. Local funding would be more sustainable to support the expected financial rewarding but also to stimulate the purchase of local materials whereby the Ministry of Health could play an important role.

Midwives, residents and gynaecologists were trained as trainers during follow up and are now acknowledged and appreciated members of the faculty. This strong group of Ethiopian trainers replaced the Dutch course director and all facilitators at the end of the project in 2020.

Educating two local course directors and local trainers gives a strong team to continue with this training in the future. Training midwifes and doctors from both health centers and hospital together, is an important factor.

A limitation of this study design was that there was no baseline assessment at the health centres. However, literature showed that availability and quality of basic and comprehensive EmONC facilities in Ethiopia is often lacking [25,26]. In order to fully understand the value of the LSTM-EmONC training (in Gondar) more robust epidemiological study designs are needed, such as randomizing health centres, stratifying participants based on for example cadre and experience and including pre- and post-intervention clinical data on maternal and neonatal mortality and morbidity and patient satisfaction. Evaluation of training could go beyond the first three levels of Kirkpatrick’s model, but for future trainings different evaluation models should be considered. Kikpatrick’s model was appropriate for evaluating this training, but the model has increasing limitations and should be used with care. As modern training programs become more personalised and user-directed, formal training is becoming less prominent and, therefore, Kirkpatrick’s model is not necessarily best suited for this new learning approach. A comparative study between the 3.5-day LSTM-EmONC course and the 21- day BEmONC course including economic evaluation, might be helpful to inform policy makers on the optimal training method. Policy makers need quantitative data such as direct obstetric case fatality rates, stillbirth rates, availability of signal functions and severe maternal morbidity to decide on the optimal training method [13]. The ‘lessons learned’ by the group of experienced facilitators described in this study could already aid in designing larger training evaluation studies.

Further studies can only be undertaken when the political conflict and the COVID-19 pandemic have proven to be under control. Publication of this article has been delayed as well due to these unpredictable circumstances.

## Conclusion

Also in Gondar, Ethiopia, the LSTM-EmONC training proved to contribute to improving knowledge and skills of health workers. Based on the data collected in Gondar and literature regarding evaluation studies of this training in different settings, we conclude that the training has the potential to increase the availability of competent skilled health personnel and to improve quality of care, which are both vital to the reduction of maternal and perinatal mortality and morbidity.

## Supporting information

S1 Checklist Inclusivity in global research. (DOCX)

S1 Text Feedback from the participants (n = 11). (DOCX)

S1 Table Level of confidence in performing after the LSTM-EmONC-training. (DOCX)

S2 Text Constraints on performing EmONC skills. (DOCX)

S1 Data Gondar training evaluation_CALC. (XLSX)
