# Supplementary material for: Evaluation of the Emergency Obstetric and Newborn Care training in Gondar, Ethiopia; a mixed methods study
Source: PLOS Glob Public Health. 2023 Sep 26;3(9):e0000889. doi: 10.1371/journal.pgph.0000889 (PMC10522022; doi:10.1371/journal.pgph.0000889)
Supplement: S1 Checklist — (DOCX) [file pgph.0000889.s001.docx]

Inclusivity in global research

**Ethical considerations, permits and authorship**

*This section is applicable to all research types.*

Provide details as to who granted permissions and/or consent for the study to take place in the Methods section of your manuscript. This should include the names of **all** ethics boards, governmental organizations, community leaders or other bodies that provided approval for the study. If individuals provided approval refer to these people by their role or title but do not list their name(s).

Page 7:

Ethical clearance was obtained from the Institutional Review Board of the University of Gondar, Ethiopia (ref no. 356/2015). Verbal consent was plenary taken at the beginning of the training and during the health center visits. All training participants (trainees and facilitators) agreed that the results of their test and evaluation would be summarized and used for analysis. All data were extracted from the questionnaires used during the training and during the health center visits. All information was anonymized.

If there were any deviations from the study protocol after approval was obtained please provide details of these changes in the Methods section of your manuscript.
Did this study involve local collaborators that are residents of the country where the research was conducted or members of the community studied? If you do not have any authors from said communities, please provide an explanation for this below.

N/A, local authors are involved.

N/A

Everyone listed as an author should meet PLOS’ criteria for authorship and all individuals who meet these criteria should be included in the author byline, rather than the acknowledgements. Authorship criteria is based on the International Committee of Medical Journal Editors (ICMJE) Uniform Requirements for Manuscripts Submitted to Biomedical Journals - for further information please see here: <https://journals.plos.org/plosone/s/authorship>.

**Human subjects research (e.g. health research, medical research, cross-cultural psychology)**

Did you obtain written informed consent from a representative of the local community or region before the research took place? How did you establish who speaks for the community? Details of written informed consent obtained from study participants should be reported separately in the Methods section of your manuscript.

We took verbal consent. All participants spoke English and/or Amharic.

How did members of the local community provide input on the aims of the research investigation, its methodology, and its anticipated outcome(s)?

The Ethiopian trainees did not provide imput. The Ethiopian trainers did provide input during the Teach the Teachers, during the meeting before start of the trainings and during the trainings itself.

When engaging with the local community, how did you ensure that the informed consent documents and other materials could be understood by local stakeholders?

N/A, the local stakeholders spoke English and/or Amharic.

Will the findings of the research be made available in an understandable format to stakeholders in the community where the study was conducted (e.g. via a presentation, summary report, copies of publications, etc.)? Please provide details of how this will be achieved.

The findings will be made available via copies of the publication. Local authors are responsible for dissemination.

**Non-human subjects research using specimens/ animals collected as part of the study, or those housed in archival collections. Examples include archaeology, paleontology, botany and zoology.**

Did the permission you obtained from a local authority to perform the study include an agreement on access to outputs and benefit sharing? This may include procedures to enable fair distribution of the benefits and resources arising from the research performed. Please include any details of Prior Informed Consent and Benefit Sharing Agreements obtained. These may be required by field-specific regulations, for example the Convention on Biological Diversity (CBD) and the associated Nagoya Protocol.

N/A

If the material used in your study was imported, please A) provide the year it was imported and B) indicate whether permits were obtained to import/export the materials used, C) provide details of any permits obtained. If this information is not available, please indicate this.

N/A

If you used archival specimens, please state how the material used in your study was acquired by the institute it is held in and provide details of any permits obtained for the original excavations/ sample collection. If this information is not available, please indicate this.

N/A

How was the potential cultural significance of the materials collected in your study to local communities considered in your research design? Were Indigenous peoples and/or local researchers and institutions involved with archaeological excavations / collection of specimens? If so, please provide a description of their involvement.

N/A

If your manuscript includes photographs of human remains please indicate whether authors obtained permission from descendants or affiliated cultural communities to do so.

N/A
