# Supplementary material for: Evaluation of the Emergency Obstetric and Newborn Care training in Gondar, Ethiopia; a mixed methods study
Source: PLOS Glob Public Health. 2023 Sep 26;3(9):e0000889. doi: 10.1371/journal.pgph.0000889 (PMC10522022; doi:10.1371/journal.pgph.0000889)
Supplement: S1 Text — (DOCX) [file pgph.0000889.s002.docx]

**S1­_Text: Feedback from the participants (n=11)**

**‘ Please extend training dates, I like the training’** (Midwife, trained in June 2015).

**‘ It’s enjoyable and skill designed training. It’s so good’** (Midwife, trained in June 2015).

**‘ Shortage of time’** (Midwife, trained in February 2016).

**‘ Continues mentoring and training for the local health centre midwifes might be helpful to maintain these practices properly’** (Medical doctor, trained in June 2015).

**‘ It’s good, lectures, skilled seniors, good time management’**. (Medical doctor, trained in June 2015)

**‘ It would be better to extend the training up to 10 days’** (Midwife, trained in June 2015).

**‘ Overall, the training was good, but it was a tight schedule. Overall, it’s better to make the training at least 5 days’** (Midwife, trained in February 2016).

**‘ The training is good, nice and interesting. Both useful knowledge and skills’** (Midwife, trained in June 2015).
