# Supplementary material for: Evaluation of the Emergency Obstetric and Newborn Care training in Gondar, Ethiopia; a mixed methods study
Source: PLOS Glob Public Health. 2023 Sep 26;3(9):e0000889. doi: 10.1371/journal.pgph.0000889 (PMC10522022; doi:10.1371/journal.pgph.0000889)
Supplement: S1 Table — (DOCX) [file pgph.0000889.s003.docx]

**S1 Table: Level of confidence in performing after the LSTM-EmONC-training**

| Skill | No confidence | Under supervision | Confident |
| --- | --- | --- | --- |
| Cardiac compression (n=6) | 0 | 3 | 3 |
| Management of shock (n=8) | 0 | 2 | 6 |
| Unconscious patient (n=7) | 0 | 2 | 5 |
| Newborn resuscitation (n=5) | 0 | 1 | 4 |
| Venous cutdown (n=5) | 4 | 0 | 1 |
| Use of partograph (n=9) | 0 | 2 | 7 |
| Vaginal breech delivery (n=8) | 1 | 2 | 5 |
| Twin delivery (n=9) | 1 | 1 | 7 |
| Shoulder dystocia (n=4) | 2 | 1 | 1 |
| Cord prolapse (n=6) | 2 | 1 | 3 |
| Active management 3^rd^ stage (n=9) | 0 | 2 | 7 |
| Manual removal placenta (n=8) | 0 | 1 | 7 |
| Antepartum haemorrhage (n=9) | 1 | 1 | 7 |
| Eclampsia (n=8) | 0 | 3 | 5 |
| Pregnancy related sepsis (n=6) | 0 | 0 | 6 |
| Vacuum extraction (n=9) | 0 | 1 | 8 |
| Septic abortion (n=5) | 0 | 0 | 5 |
| Manual vacuum aspiration (n=1) | 0 | 0 | 1 |
| Neonatal sepsis (n=2) | 0 | 0 | 2 |
| Premature care (n=5) | 0 | 0 | 5 |
| Performing episiotomy (n=7) | 0 | 1 | 6 |
| Suturing episiotomy (n=9) | 0 | 2 | 7 |
| Suturing perineal tears (n=6) | 1 | 2 | 3 |
| Suturing cervical tears (n=4) | 1 | 2 | 1 |
| Total | 13 | 30 | 112 |

No confidence: number of participants that felt no confidence to perform the skill.

Under supervision: number of participants that only feel confident to perform skill under supervision.

Confident: number of participants that feel confident to perform the skill alone.
