# Supplementary material for: Evaluation of the Emergency Obstetric and Newborn Care training in Gondar, Ethiopia; a mixed methods study
Source: PLOS Glob Public Health. 2023 Sep 26;3(9):e0000889. doi: 10.1371/journal.pgph.0000889 (PMC10522022; doi:10.1371/journal.pgph.0000889)
Supplement: S2 Text — (DOCX) [file pgph.0000889.s004.docx]

**S2­­_Text: Constraints on performing EmONC skills.**

‘ **Even when the vacuum is available, it’s not functional’** (Midwife, trained in June 2015).

**‘ After the training I’m solving different types of cases and manage more complicated cases.**

(Midwife, trained in June 2015).

**‘ Often there is a lack of magnesium sulphate in this health centre’** (Midwife, trained in June 2015).

**‘ This health centre lacks medical equipment, infrastructure and admission beds’** (Midwife, trained in June 2015).

**‘ This health centre lacks magnesium sulphate, vacuum and MVA’** (Midwife, trained in June

2015).

**‘ I need more training to feel confident, besides there is no follow-up sheet for antenatal care and**

**partograph, also lack of magnesium sulphate’** (Midwife, trained in June 2015).
